# A note on the use of the generalized odds ratio in meta-analysis of association studies involving bi- and tri-allelic polymorphisms

**DOI:** 10.1186/1756-0500-4-172

**Published:** 2011-06-06

**Authors:** Tiago V Pereira, Regina C Mingroni-Netto

**Affiliations:** 1 Centro de Estudos do Genoma Humano, Departamento de Genética e Biologia Evolutiva, Instituto de Biociências, Universidade de São Paulo, São Paulo

## Abstract

**Background:**

The generalized odds ratio (GOR) was recently suggested as a genetic model-free measure for association studies. However, its properties were not extensively investigated. We used Monte Carlo simulations to investigate type-I error rates, power and bias in both effect size and between-study variance estimates of meta-analyses using the GOR as a summary effect, and compared these results to those obtained by usual approaches of model specification. We further applied the GOR in a real meta-analysis of three genome-wide association studies in Alzheimer's disease.

**Findings:**

For bi-allelic polymorphisms, the GOR performs virtually identical to a standard multiplicative model of analysis (e.g. per-allele odds ratio) for variants acting multiplicatively, but augments slightly the power to detect variants with a dominant mode of action, while reducing the probability to detect recessive variants. Although there were differences among the GOR and usual approaches in terms of bias and type-I error rates, both simulation- and real data-based results provided little indication that these differences will be substantial in practice for meta-analyses involving bi-allelic polymorphisms. However, the use of the GOR may be slightly more powerful for the synthesis of data from tri-allelic variants, particularly when susceptibility alleles are less common in the populations (≤10%). This gain in power may depend on knowledge of the direction of the effects.

**Conclusions:**

For the synthesis of data from bi-allelic variants, the GOR may be regarded as a multiplicative-like model of analysis. The use of the GOR may be slightly more powerful in the tri-allelic case, particularly when susceptibility alleles are less common in the populations.

## Findings

The generalized odds ratio (GOR) was recently suggested as a model-free measure of effect that might overcome the problem of a genetic model misspecification in meta-analyses of association studies [1]. In the context of case-control genetic association studies for a binary trait and under assumption of random sampling, the GOR measures the probability that a case has a higher mutation load (i.e. a larger number of high-risk alleles) than a control divided by the probability that a control has a higher mutation load than a case.

In this note, we highlight advantages and limitations of the use of the GOR as a measure of effect in meta-analyses of bi- and tri-allelic polymorphisms through simulation. Our results are further complemented by a re-analysis of a real meta-analysis of three genome-wide association studies covering >311,000 bi-allelic markers in Alzheimer's disease.

## Results

### Performance of the GOR in the bi-allelic model

#### Type-I error rates

Type-I error rates obtained from meta-analyses employing the GOR as a summary effect size are comparable to the multiplicative and dominant models of analysis (Table 1).

**Table 1:** Type-I error rates (%) for the bi-allelic case according to different genetic models of analysis and heterogeneity (τ^2 ^) for α = 5%

	**τ**^**2 **^**= 0**					**τ**^**2 **^**= 0.025**					**τ**^**2 **^**= 0.05**				
			
**Model of analysis**	**Allelic**	**LAT**	**GOR**	**Domi**	**Rece**	**Allelic**	**LAT**	**GOR**	**Domi**	**Rece**	**Allelic**	**LAT**	**GOR**	**Domi**	**Rece**
		
**No. of Studies**	**Fixed-effects, MAF = 10%**														
2	4.82	4.82	4.82	4.86	2.30	12.34	12.28	13.44	13.60	2.54	16.98	16.80	18.72	18.86	2.68
5	5.34	5.36	5.12	5.16	2.10	11.34	11.22	12.80	12.90	2.42	17.88	17.96	19.80	20.16	2.48
7	4.80	4.80	4.94	4.78	1.76	11.46	11.30	12.78	12.88	1.98	17.94	17.78	19.66	20.08	2.72
10	5.00	5.00	5.02	5.00	1.92	11.98	11.80	13.26	13.42	2.12	17.72	17.72	19.60	20.02	2.76
20	4.86	4.80	5.06	5.18	1.68	11.52	11.36	12.36	12.64	2.02	17.72	17.60	19.28	19.32	2.62
30	4.96	4.92	4.96	5.02	1.94	11.94	11.78	12.84	13.06	2.04	18.52	18.40	20.18	20.40	2.38
**No. of Studies**	**Random-effects, MAF = 10%**														
2	3.76	3.76	3.66	3.60	2.24	8.38	8.28	8.72	8.84	2.44	10.54	10.62	11.58	11.56	2.66
5	4.14	4.10	3.92	3.84	1.90	6.90	6.78	7.48	7.50	2.20	8.76	8.68	9.08	9.30	2.28
7	3.70	3.70	3.74	3.76	1.66	7.02	6.92	7.14	7.22	1.88	8.34	8.34	8.66	8.68	2.48
10	3.88	3.84	4.04	4.16	1.80	7.14	7.02	7.52	7.42	1.98	7.30	7.20	7.56	7.58	2.64
20	4.12	4.14	4.42	4.42	1.66	6.06	6.04	6.32	6.36	1.92	6.86	6.88	6.88	6.78	2.42
30	4.16	4.16	4.34	4.32	1.88	5.82	5.80	5.76	5.84	1.96	6.24	6.34	6.06	5.88	2.24
**No. of Studies**	**Fixed-effects, MAF = 40%**														
2	4.82	4.72	4.84	4.96	4.70	9.82	9.66	9.74	12.32	13.78	15.62	15.40	15.60	19.82	22.58
5	4.98	4.86	4.88	4.68	4.90	10.42	10.38	10.46	12.42	14.60	15.66	15.42	15.84	20.14	22.52
7	5.22	4.98	4.82	4.56	5.08	10.34	10.18	10.62	12.92	15.04	16.26	16.16	16.26	20.44	21.58
10	5.02	4.98	4.86	4.68	5.26	11.42	11.26	11.08	13.22	14.94	15.64	15.42	15.74	20.46	22.38
20	5.18	5.12	5.06	4.94	5.14	10.80	10.62	10.84	13.82	14.76	16.22	15.98	16.34	20.36	23.52
30	4.60	4.60	4.66	4.62	4.56	11.34	11.28	11.44	13.88	15.22	16.56	16.48	16.88	20.26	22.64
**No. of Studies**	**Random-effects, MAF = 40%**														
2	3.82	3.76	3.82	4.04	3.58	6.92	6.86	7.00	8.42	9.10	9.54	9.66	9.86	12.34	13.58
5	3.82	3.70	3.72	3.76	3.60	6.18	6.16	6.02	6.72	7.86	8.50	8.40	8.34	9.60	9.28
7	3.94	3.80	3.58	3.46	3.96	6.08	6.02	6.30	6.78	7.68	8.02	7.98	7.92	9.20	8.46
10	3.94	3.90	3.88	3.48	4.00	6.20	6.20	6.32	6.96	6.90	7.10	7.08	7.30	7.98	7.40
20	4.14	4.12	4.16	4.24	4.16	5.90	5.88	6.16	6.74	6.02	6.96	6.96	7.06	6.88	6.26
30	3.90	3.86	3.90	3.92	3.74	6.74	6.66	7.04	6.72	6.16	6.88	6.98	6.84	6.92	5.32

Allelic, per-allele odds ratio. LAT, log-additive trend. GOR, generalized odds ratio. Domi, dominant. Rece, recessive. MAF, minor allele frequency. Results are based on 5,000 simulations. τ^2^, between-study variance. The size for each study was randomly sampled from a uniform distribution on the interval [500-1000] and split equally into cases and controls (i.e. case to control ratio = 1).

#### Power

**Figure 1:**
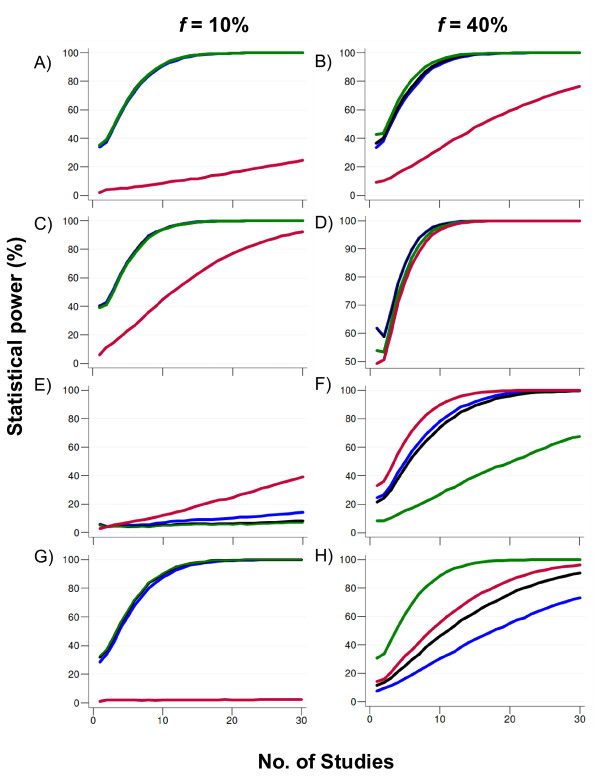
**Power (at α = 5%) for the bi-allelic case for a representative scenario of a variant with modest effect (OR = 1.3) following distinct modes of action (A-B, dominant; C-D, multiplicative, E-F, recessive, and G-H, over-dominant) under moderate heterogeneity (τ^2 ^= 0.025)**. The sample size for each study was randomly sampled from a uniform distribution on the interval [500-1000] and split equally into cases and controls (i.e. case to control ratio = 1). Color lines depict power estimates under different models of analysis: green (dominant), blue (per-allele odds ratio), red (recessive) and black (generalized odds ratio). Results for the log-additive trend were omitted because the striking similarity with the results of the per-allele odds ratio. Results are based on 5,000 replications under a random-effects model (DerSimonian-Laird method). *f*, allelic frequency. Scenarios with alternative magnitudes of heterogeneity or use of a fixed-effects model yielded qualitatively identical results.

Compared to the use of multiplicative approaches, the power to detect variants with a dominant model of action was typically only slightly higher for meta-analyses using the GOR as summary estimate. For variants following a multiplicative pattern of action, all non-recessive models of analysis were highly comparable. Interestingly, the largest differences observed among the per-allele, log-additive trend (LAT) and the GOR were found in true recessive and over-dominant models, where the performance of the GOR is slightly inferior for the former, but reasonable better for the latter (Figure 1).

#### Bias in the estimated statistical heterogeneity (τ^2^)

**Figure 2:**
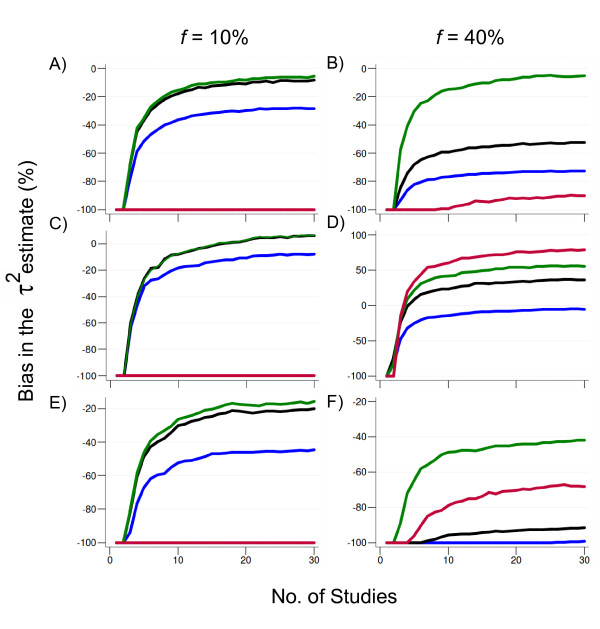
**Bias (%) in the between-study variance (τ^2 ^) estimate (statistical heterogeneity) for the bi-allelic case for a representative scenario of a variant with modest effect (OR = 1.3) following distinct modes of action (A-B, dominant; C-D, multiplicative, E-F, over-dominant) under moderate heterogeneity (τ^2 ^= 0.025)**. The sample size for each study was randomly sampled from a uniform distribution on the interval [500-1000] and split equally into cases and controls (i.e. case to control ratio = 1). Color lines depict bias estimates under different models of analysis: green (dominant), blue (per-allele odds ratio), red (recessive) and black (generalized odds ratio). Results for the log-additive trend model of analysis were omitted because the striking similarity with the results of the per-allele odds ratio. Scenarios with a genuine variant acting recessively are not displayed for simplicity, since all models of analysis (except the recessive) are unable to capture the underlying τ^2 ^even when the frequency of risk alleles is high. Scenarios with alternative magnitudes of heterogeneity yielded qualitatively identical results. Results are based on the median value from 5,000 replications. *f*, allelic frequency.

Compared to both per-allele and LAT approaches, the median bias in τ^2 ^obtained by the GOR is typically lower in scenarios where the genetic variant is less common in the populations (e.g. minor allele frequency [MAF] = 10%) and acts either dominantly or multiplicatively. For the latter model of action, bias is slightly positive. In addition, for common markers (MAF = 40%) following a dominant model of action, the GOR provides less biased τ^2 ^estimates compared to the specification of a multiplicative model. Importantly, for a common variant (MAF = 40%) acting multiplicatively, meta-analyses using the GOR as an effect size provide upwardly biased estimates of τ^2 ^compared to true underlying average increment in the between-study variance per additional copy of the risk allele (Figure 2). This upward bias in the estimated statistical heterogeneity is also found in both dominant and recessive models of analysis.

#### Bias in the estimated genetic effect size

The GOR provides nearly unbiased summary effects for less common variants (MAF = 10%) acting dominantly, regardless of the meta-analytical model and τ^2^. Conversely, when the variant follows a multiplicative model of action and is common (MAF = 40%), GOR-based meta-analyses overestimate the true underlying increase in the effect size per additional copy of the risk allele (on average 20%) [Additional file 1: Supplementary tables S1-S2].

### Performance of the GOR in the tri-allelic model

#### Type-I error rates

The performance of each model of analysis depends on the underlying between-study variability, allele frequencies and meta-analytical model, but type-I error rates for LAT- and GOR-based meta-analyses are comparable, whereas false discoveries tend to be higher for the per-allele approach when statistical heterogeneity is present (i.e. τ^2 ^>0). However, the extent of these differences is smaller in random-effects calculations [Additional file 1: Supplementary tables S3-S4].

#### Power: two alleles acting on the same direction

**Figure 3:**
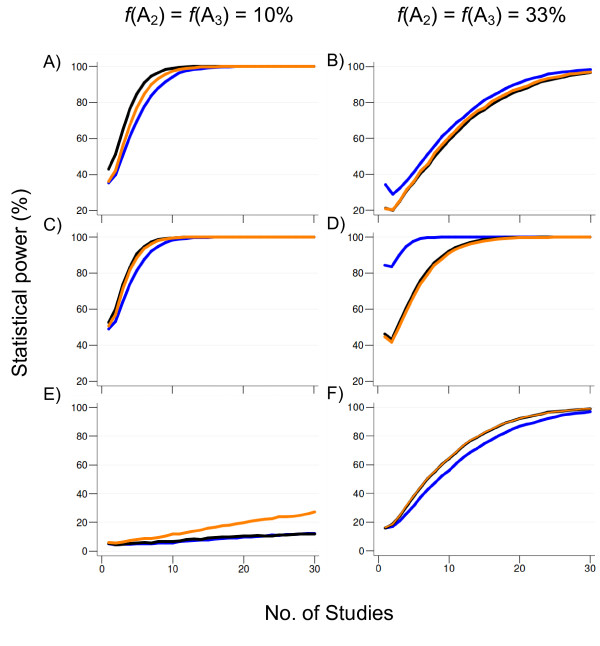
**Power (at α = 5%) for the tri-allelic case for a representative scenario of two alleles (*A*_2 _and *A*_3_) acting on the same direction with modest effect (OR = 1.3) following distinct modes of action (A-B, dominant; C-D, multiplicative, E-F, recessive) under moderate heterogeneity (τ^2 ^= 0.025)**. The sample size for each study was randomly sampled from a uniform distribution on the interval [500-1000] and split equally into cases and controls (i.e. case to control ratio = 1). Color lines depict power estimates under different models of analysis: orange (log-additive trend), blue (Dunn-Šidák-corrected per-allele odds ratio) and black (generalized odds ratio). Results are based on 5,000 replications under a random-effects model (DerSimonian-Laird method). *f*, allelic frequency. Scenarios with alternative magnitudes of heterogeneity or use of a fixed-effects model yielded qualitatively identical results.

When at least one of the risk-alleles is less common in the populations (*f *= 10%), and both exhibit either a dominant or multiplicative mode of action, power obtained by using the GOR as a summary effect is higher than that provided by either the per-allele or LAT approaches (Figure 3).

#### Power: two alleles acting on opposite directions

When prior evidence on the direction of the effects of the susceptibility alleles is available, similar power is achieved with the use of the per-allele, LAT and GOR, regardless of the meta-analytical model, *f *and statistical heterogeneity [Additional files 1: Supplementary tables S5-S7].

**Figure 4:**
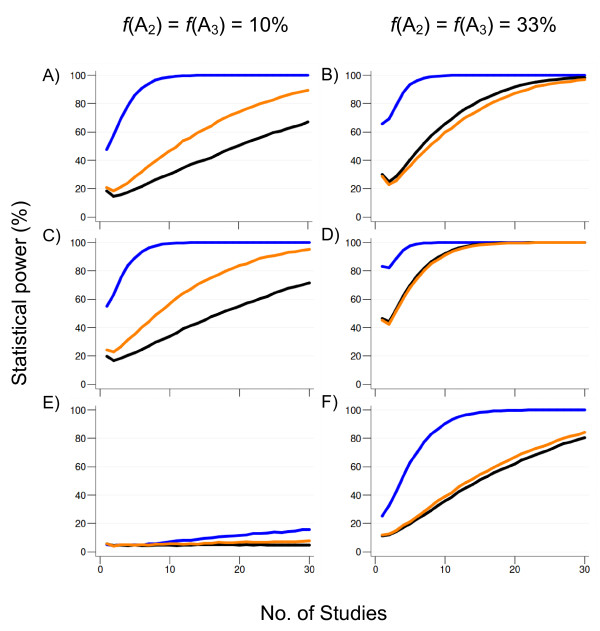
**Power (at α = 5%) for the tri-allelic case for a representative scenario of two alleles (*A*_2 _and *A*_3_) acting in opposite directions (*A*_2 _is protective, whereas *A*_3 _is the susceptibility allele) with modest effect (OR = 0.77 for allele *A*_2 _and OR = 1.3 for allele *A*_3_) following distinct modes of action (A-B, dominant; C-D, multiplicative, E-F, recessive) under moderate heterogeneity (τ^2 ^= 0.025)**. The sample size for each study was randomly sampled from a uniform distribution on the interval [500-1000] and split equally into cases and controls (i.e. case to control ratio = 1). Color lines depict power estimates under different models of analysis: orange (log-additive trend), blue (Dunn-Šidák-corrected per-allele odds ratio) and black (generalized odds ratio). Results are based on 5,000 replications under a random-effects model (DerSimonian-Laird method). *f*, allelic frequency. Scenarios with alternative magnitudes of heterogeneity or use of a fixed-effects model yielded qualitatively identical results.

On the other hand, when no prior evidence on the direction of effects is available (e.g. initial screenings), the per-allele model of analysis displays a superior performance compared to the use of either the LAT- or GOR-based approaches. In particular, compared to both GOR and LAT approaches, the gain in power for meta-analyses using the per-allele OR may range from 1.5- to 10-fold depending on the number of combined studies (Figure 4).

#### Power: when only one allele displays a significant effect

Power is comparable among the GOR, LAT and per-allele odds ratio when only one allele displays a significant effect. This is specially true when the high-risk allele is less common in the populations (*f *= 10%), particularly when *f *(A_2_) = *f *(A3) = 10%. Overall, for common variants acting multiplicatively, the best performance is achieved with both GOR and LAT. When the risk allele is either recessive or dominant and is common, the best approach may depend on the frequency of the remaining alleles, but power is comparable among the three tested approaches whenever *f *(A_2_) ≅ *f *(A3) [Additional file 1: Supplementary tables S8-S10].

### Real application

Results for the seven "top hits" variants associated with late-onset Alzheimer's disease are presented in Table 2. As expected, the largest association signal arose from the variant rs41377151, located at the 3' end of the apolipoprotein C-I (*APOC1*) gene within the Apolipoprotein E (*APOE)*/*APOC1 *gene cluster on chromosome 19q13.3. This polymorphism is only 10.9 kb away from rs7412 variant (Arg176Cys) [2], which is one of the alleles that dictate the *APOE *ε status [3]. In addition, the remaining signals are also commensurate with results from previous [4] and more recent, large investigations [2,5,6].

**Table 2:** Summary results according to different models of analysis for the seven strongest association signals obtained by a meta-analysis of three independent genome-wide association studies in Alzheimer's disease (TGen data sets, Reiman dise *et al*. 2007)

SNP	Chr	Gene	MAF^a^	Model of analysis	OR (95% IC)		*P*-value		P(*Q*)	*I*^2^
		
					Fixed	Random	Fixed	Random		
								
rs41377151	19q13.32	*APOC1*(3'region)	0.30	Allelic	3.00 (2.50-3.59)	3.15 (2.20-4.53)	2.14 × 10^-32^	4.65 × 10^-10^	0.05	67
				LAT	2.94 (2.43-3.56)	3.06 (2.12-4.43)	1.62 × 10^-28^	2.79 × 10^-9^	0.05	66
				GOR	3.44 (2.79-4.25)	3.64 (2.38-5.57)	1.73 × 10^-30^	2.73 × 10^-9^	0.04	68
				Domi	3.51 (2.81-4.40)	3.78 (2.35-6.08)	3.33 × 10^-28^	4.19 × 10^-8^	0.04	70
				Rece	5.51 (3.32-9.14)	5.51 (3.32-9.14)	4.31 × 10^-11^	4.31 × 10^-11^	0.59	0
rs17330779^b^	7q31	*NRCAM *(intron)	0.10	**Allelic**	0.53 (0.41-0.69)	0.53 (0.41-0.69)	1.61 × 10^-6^	1.61 × 10^-6^	0.42	0
				**LAT**	0.50 (0.38-0.65)	0.50 (0.38-0.65)	5.96 × 10^-7^	5.96 × 10^-7^	0.38	0
				**GOR**	0.49 (0.37-0.65)	0.49 (0.37-0.65)	4.72 × 10^-7^	4.72 × 10^-7^	0.42	0
				**Domi**	0.49 (0.37-0.65)	0.49 (0.37-0.65)	5.01 × 10^-7^	5.01 × 10^-7^	0.44	0
				**Rece**	-	-	-	-	-	-

rs10824310^b^	10q11.23	*PRKG1 *(intron)	0.06	**Allelic**	0.47 (0.35-0.64)	0.47 (0.35-0.64)	2.11 × 10^-6^	2.11 × 10^-6^	0.63	0
				**LAT**	0.45 (0.33-0.62)	0.45 (0.33-0.62)	1.41 × 10^-6^	1.41 × 10^-6^	0.56	0
				**GOR**	0.44 (0.31-0.61)	0.44 (0.31-0.61)	7.37 × 10^-7^	7.37 × 10^-7^	0.67	0
				**Domi**	0.43 (0.31-0.60)	0.43 (0.31-0.60)	7.04 × 10^-7^	7.04 × 10^-7^	0.68	0
				**Rece**	-	-	-	-	-	-

rs12162084	16	Unknown	0.16	**Allelic**	0.61 (0.50-0.75)	0.61 (0.50-0.75)	2.28 × 10^-6^	2.28 × 10^-6^	1.00	0
				**LAT**	0.59 (0.48-0.73)	0.59 (0.48-0.73)	1.56 × 10^-6^	1.56 × 10^-6^	0.99	0
				**GOR**	0.56 (0.45-0.71)	0.56 (0.45-0.71)	9.50 × 10^-7^	9.50 × 10^-7^	0.94	0
				**Domi**	0.56 (0.44-0.70)	0.56 (0.44-0.70)	1.15 × 10^-6^	1.15 × 10^-6^	0.84	0
				**Rece**	0.55 (0.26-1.15)	0.52 (0.17-1.60)	1.13 × 10^-1^	2.55 × 10^-1^	0.22	34

rs7077757	10q25.2	*RBM20 *(intron)	0.21	**Allelic**	0.64 (0.53-0.77)	0.64 (0.53-0.77)	2.45 × 10^-6^	2.45 × 10^-6^	0.92	0
				**LAT**	0.62 (0.51-0.76)	0.62 (0.51-0.76)	2.10 × 10^-6^	2.10 × 10^-6^	0.99	0
				**GOR**	0.59 (0.47-0.73)	0.59 (0.47-0.73)	1.34 × 10^-6^	1.34 × 10^-6^	0.99	0
				**Domi**	0.57 (0.46-0.72)	0.57 (0.46-0.72)	1.85 × 10^-6^	1.85 × 10^-6^	0.94	0
				**Rece**	0.58 (0.34-1.00)	0.57 (0.30-1.10)	4.81 × 10^-2^	9.25 × 10^-2^	0.29	20

rs10747758	12q14.2	*OR6U2P*	0.37	**Allelic**	0.69 (0.58-0.81)	0.69 (0.58-0.81)	3.97 × 10^-6^	3.97 × 10^-6^	0.50	0
				**LAT**	0.68 (0.57-0.80)	0.68 (0.57-0.80)	4.01 × 10^-6^	4.01 × 10^-6^	0.53	0
				**GOR**	0.62 (0.51-0.76)	0.62 (0.51-0.76)	2.04 × 10^-6^	2.04 × 10^-6^	0.52	0
				**Domi**	0.58 (0.46-0.73)	0.58 (0.46-0.73)	3.40 × 10^-6^	3.40 × 10^-6^	0.67	0
				**Rece**	0.66 (0.48-0.90)	0.66 (0.48-0.90)	9.70 × 10^-3^	9.70 × 10^-3^	0.54	0

rs2517509^b^	6p21.33	*HCG22*	0.03	**Allelic**	3.22 (1.94-5.34)	3.22 (1.94-5.34)	5.77 × 10^-6^	5.77 × 10^-6^	0.45	0
				**LAT**	3.27 (1.96-5.46)	3.30 (1.93-5.63)	5.85 × 10^-6^	1.27 × 10^-5^	0.35	4.7
				**GOR**	3.31 (1.97-5.57)	3.33 (1.95-5.70)	6.58 × 10^-6^	1.14 × 10^-5^	0.36	3.3
				**Domi**	3.31 (1.96-5.58)	3.32 (1.94-5.69)	7.53 × 10^-6^	1.19 × 10^-5^	0.36	2.8
				**Rece**	-	-	-	-	-	-

^a^MAF, study size-weighted average minor allelic frequency. OR, odds ratio. 95% CI, 95% confidence intervals. Allelic, per-allele odds ratio. LAT, log-additive trend. GOR, generalized odds ratio. Domi, dominant. Rece, recessive. *P*(*Q*), *P*-value for the Cochran *Q*-statistic. Chr, chromosome. *APOC1*, Apolipoprotein C1. *NRCAM*, neuronal cell adhesion molecule. *PRKG1*, protein kinase, cGMP-dependent, type I. *RBM20*, RNA binding motif protein 20. *OR6U2P*, olfactory receptor, family 6, subfamily U, member 2 pseudogene. *HCG22*, HLA complex group 22. ^b^For the rs17330779, rs10824310 and rs2517509 variants, analyses under a recessive model were not possible due to the small minor allelic frequency (i.e. sparse data). Results are based on crude odds ratios (i.e. individual study estimates were not adjusted for gender and *APOE *status).

**Figure 5:**
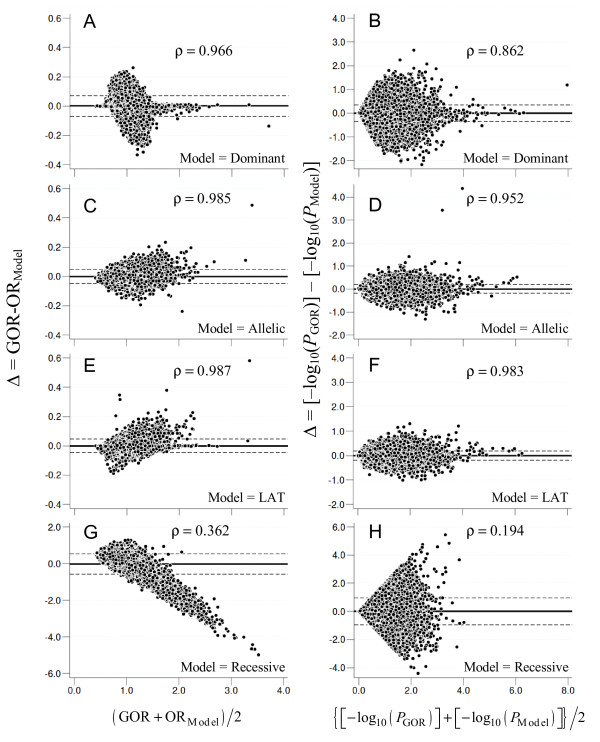
**Bland-Altman plots depicting agreement between random-effects model-based summary odds ratios (Panels A, C, E and G) and *P*-values (Panels B, D, F and H) obtained by traditional genetic models of analysis and the generalized odds ratio (GOR)**. *P*-values are given on a -log10 scale. Dashed lines represent 95% confidence limits of agreement, computed as Δ ± 1.96(standard deviation of Δ). Within each panel, the spearman coefficient (ρ) is shown, and the summarizes the correlation between estimates obtained from GOR and other models of analysis.

In agreement with our simulation-based results, plots of summary ORs and *P*-values (Figure 5) based on real data suggest a good concordance between GOR and both LAT and per-allele approaches, followed by the dominant and recessive models, respectively.

## Discussion

The GOR was suggested as a model-free approach for the synthesis of genetic association studies. The rational is that the GOR provides more flexibility for the true underlying genetic effect to describe the difference between two cumulative distribution functions of the latent variables, particularly when the assumption of proportional odds is violated. Furthermore, an additional advantage is that this ordinal measure of association is easily interpretable in practice [1].

Recent meta-analyses have applied the GOR claiming that this might be considered a different genetic model or an independent approach compared to the specification of traditional genetic model of analysis [7,8]. However, here we show that, since the GOR inherently assumes an ordinal mutation load (e.g. 1, 2 and 3 for genotypes *A*_1_*A*_1_, *A*_1_*A*_2_, *and A*_2_*A*_2_, respectively), this measure of assocation performs like a multiplicative model of analysis for bi-allelic polymorphisms. For diallelic variants, our simulations show that GOR-based results are highly correlated to those obtained by both LAT and per-allele ORs, resulting in similar type-I error rates and power compared to these traditional multiplicative models of analysis. In addition, a real meta-analysis of three GWAs in Alzheimer's disease indicates that limited. For example, under a fixed-effects framework and assumption of a threshold of *P*<10^-5 ^(probably realistic due to the small samples sizes available), the total number of markers considered promising for further replication [9] would be 10, 13, 13, 14 and two for the per-allele, LAT, GOR, dominant and recessive approaches, respectively. Under a random-effects model, the correspondent numbers would be two for the recessive model and 8 for the remaining approaches.

Nonetheless, other important considerations in meta-analysis of genetic association studies involving bi-allelic polymorphism are biases in the estimated effect size [10] and heterogeneity [11]. In this respect, the most negative aspect of using the GOR as a measure of association in practice is that this measure provides inflated effects for bi-allelic variants following a multiplicative model of action. Although this inflation may be only mild for less common markers (i.e. median bias of ~5% for variants with MAF = 10%), the average upward bias in the observed genetic effect augments with increasing MAFs, reaching up to 20% for MAFs around 40%.

On the other hand, our data showed that the use of the GOR may be advantageous in meta-analyses involving tri-allelic polymorphisms as long as genotypes can be correctly ordered in terms of mutation load. In fact, a reasonable gain in power in the order of 2 to 15% may be achieved for the detection of association signals from variants with small frequencies (e.g. f ~10%) compared to the use of per-allele or LAT odds ratios. The observation that higher power might be obtained with GOR in scenarios with a larger number of alleles of low frequency may serve as hypothesis-generating information to extent the use of the GOR to meta-analysis of different types of genetic variants. For example, a special case might the use of the GOR in meta-analysis of structural variants such as copy-number variations (CNVs), which tend to exhibit a substantial number of alleles, yielding a correspondent large number of possible genotype categories [12]. Since the GOR handles categories with zero counts [13], and a different number of genotypes may be considered per study (for instance, in the case of specific allele sizes in some populations), the properties of the GOR in meta-analysis of CNVs is a topic worth of further investigation.

In summary, although there are differences in the statistical properties among the investigated approaches for bi-allelic variants, the absolute magnitude of these differences may be actually small and likely to be of very limited practical significance. An exception might be the use of the GOR in meta-analyses involving tri-allelic polymorphisms with less common alleles, since GOR uses of the complete genotypic distribution (e.g. the GOR less affected by zero cells). For these scenarios, the use of the GOR as a measure of effect may be slightly more powerful than traditional measures. However, the performance of GOR-based meta-analyses will depend on some knowledge about the direction of the effects when there are two alleles modulating the risk of disease in opposite directions.

## Material and methods

### Simulation procedures and scenarios

We simulated meta-analyses of association studies using approaches that rely on multinomial distributions described in detail elsewhere (autosomal markers) [9,10]. Hardy-Weinberg equilibrium is assumed to hold for the whole population, whereas the susceptibility alleles are considered the causal variants or surrogate markers in tight linkage disequilibrium (*r*^2 ^= 1.0). For the bi-allelic case, we simulated data assuming the susceptibility variant *A*_2 _(minor allele) and non-risk allele *A*_1_.

Under a three-allele model, we denote *A*_1_, *A*_2 _and *A*_3 _as the possible alleles with frequencies *f*(*A_1_*), *f*(*A_2_*) and *f*(*A_3_*^)^, respectively, yielding six possible distinct genotypes (*A*_1_*A*_1_, *A*_1_*A*_2_, *A*_1_*A*_3_, *A*_2_*A*_2_, *A*_2_*A*_3 _and *A*_3_*A*_3_).

For each possible combination of the parameters presented in Table 3 we considered meta-analyses that included two up to 30 studies (case-to-control ratio of 1:1).

**Table 3:** Parameters and simulated scenarios (trait prevalence = 10%).

*True underlying genetic model*	*OR*	*f*	τ^2^	*N per study*	*Genetic model of analysis*
Null	1.0		0 (homogeneity)		GOR
		0.10			LAT
		0.33	0.025 (mild heterogeneity)	500-1000	Per-allele
		0.40			Dominant
Dominant Multiplicative Recessive Over-dominant	1.3 (modest effect)		0.05 (strong heterogeneity)		Recessive

OR, true underlying odds ratio. *f*, allelic frequency for the risk allele. τ^2^, between-study variance. *N*, number of participants per study. GOR, generalized odds ratio. LAT, log-additive trend. The size for each study was randomly sampled from a uniform distribution on the interval [500-1000] and split equally into cases and controls (i.e. case to control ratio = 1).

For the tri-allelic case, three possible scenarios were considered: (*i*), among the alleles, two were susceptibility variants (e.g. both increase the susceptibility for the trait with the same magnitude), (*ii*) two alleles were associated with the trait, but in opposite directions (i.e. one increases, while the other decreases the risk for the trait in a similar magnitude) and (*iii*) only one out of the three alleles displays significant effects on the trait. We further assumed that the mechanism of action is similar for both alleles when there are two alleles with genuine effects on the trait (e.g. both act multiplicatively, or both act dominantly, and so forth). For scenarios with two alleles modulating the risk of disease, two additional situations of practical interest were investigated: (*ii-a*) the two alleles are associated with the susceptibility of disease in opposite directions and investigators have *no prior *evidence on the direction of these effects (e.g. initial agnostic screenings) and (*ii*-*b*) two alleles are associated with the susceptibility of disease in opposite directions, but investigators *posses prior *evidence on the direction of the effects (e.g. meta-analyses from the literature). For consistency, allele *A*_2 _is coined to be the protective variant, whereas allele *A*_3 _is the susceptibility one in these scenarios.

### Bi-allelic polymorphisms

#### Assessment of bias

The percentage bias was computed as  and  for genetic effect sizes and between-study variance, respectively, where  is the (average) observed summary effect, μ is the true average genetic effect across population-specific genetic effects, τ^2 ^is the true between-study variance and  is the method-of-moments-based estimate of τ^2^. Both  and μ are captured as the natural logarithm of the odds ratio (Table 3). Use of alternative bias estimators (e.g. mean squared error) yielded qualitatively analogous results (data not shown).

### Tri-allelic polymorphisms

Meta-analyses involving three-allele polymorphisms may rely on a diversity of approaches to summarize effects across studies. However, because the assumption of multiplicative effects yields, on average, the lowest rates of false-positive results in bi-allelic markers [9,10], we compared the GOR to two approaches that assume a multiplicative mode of action: the per-allele OR, which yields to three correlated odds ratios (OR[A_3 _vs A_1_], OR[A_3 _vs A_2_] and OR[A_2 _vs A_1_]) and the log-additive trend approach.

### The generalized odds ratio

For a binary trait (e.g. case-control studies), GOR measures the probability that a randomly sampled case has a genotype with a higher mutation load (i.e. a larger number of high-risk alleles) than a randomly sampled control divided by the probability that a randomly sampled case has a genotype with lower mutation load than a randomly sampled control [1].

The GOR for a binary trait and an *m*-allelic variant can be computed as [13]:(1)

where *J *is the total number of genotypes (categories) given the number of alleles, i.e., *J *= *m*(*m*+1)/2, *m *is the number of alleles,  (i.e. the proportion of the subjects with genotype *j*, for *j *= 1,..,*J*, in which the higher the value of *j*, the higher the mutation load) in the group *i *(*i *= 0 or 1 for controls and cases, respectively). In the present investigation, the large-sample variance for GOR was computed from the asymptotic standard error of the Goodman-Kruskal γ [1]. Stata and R codes to compute the GOR and its large-sample variance are available from the first author upon request.

### Mutational load order

The order of the *j*th genotypic category (i.e. mutational load) for the GOR and log-additive trend is anticipated to impact statistical power. Hence, for the situation *ii*-a (initial agnostic screenings), we set  as genotypic order and  for situation *ii*-b (meta-analyses from the literature with prior information on the direction of effects).

### Assessment of power and type-I error

Empirical power and type-I error rates (i.e. false-positive discoveries) were computed as the proportion of simulations that gave a two-sided *P*-value < 5%. Because there are three correlated OR estimates for the tri-allelic case for the per-allele model, we corrected the α level using the Dunn-Šidák procedure. Specifically, power and type-I error rates for the per-allele model (tri-allelic case) were computed as the proportion of the simulations that gave one or more *P*-values < α*_corrected_*, where .

### Real application

We compared results based on the GOR as a summary effect to those obtained by usual approaches of model specification in a real meta-analysis of three independent genome-wide studies in late-onset Alzheimer's disease. After standard control measures, a total of 311,915 bi-allelic polymorphisms were scored in 1411 participants (961 cases and 560 controls). Detailed description on the samples, genotyping platforms and diagnostics criteria are available elsewhere [4]. Results from individual studies were corrected for residual inflation of the test statistic using genomic control methods [14].

### Meta-analysis methods

Meta-analyses were carried out under both fixed- and random-effects models, represented by the general inverse-variance and DerSimonian-Laird methods, respectively [15,16]. For the real application, statistical heterogeneity was test using the Cochran's *Q *test [11], and quantified using the *I*^2 ^index [17].

All simulations were performed in Stata 11.1 package (Stata Corporation), whereas the meta-analysis of real data sets were carried out in PLINK [18].

## Competing interests

The authors declare that they have no competing interests.

## Authors' contributions

TVP carried out the computational experiments, tabulated the data and drafted the manuscript. TVP and RCMN conceived the study. RCMN participated in its design and coordination and helped to draft the manuscript. Both authors read and approved the final manuscript.

## Supplementary Material

Additional file 1 **Supplementary tables S1 through S10**. Click here for file
