# Supplementary material for: A note on the use of the generalized odds ratio in meta-analysis of association studies involving bi- and tri-allelic polymorphisms
Source: BMC Res Notes. 2011 Jun 6;4:172. doi: 10.1186/1756-0500-4-172 (PMC3146434; doi:10.1186/1756-0500-4-172)
Supplement: Additional file 1 — Supplementary tables S1 through S10. [file 1756-0500-4-172-S1.PDF]

Supplementary tables

Table S1. Median percentage bias in the estimated effect size in random-effects meta-analyses using different models of analysis to detect a modest effect (OR = 1.3) of a bi-allelic polymorphism with MAF = 10% that follows distinct modes of action.

	τ^2 =0					τ^2 =0.025					τ^2 =0.05				
**Model of analysis**	**Allelic**	**LAT**	**GOR**	**Domi**	**Rece**	**Allelic**	**LAT**	**GOR**	**Domi**	**Rece**	**Allelic**	**LAT**	**GOR**	**Domi**	**Rece**
**No. of Studies**								**Dominant**							
2	-9.5	-8.3	-0.2	0.7	-17.6	-10.2	-9.0	-0.1	1.2	-7.3	-11.1	-9.2	-1.1	0.5	-16.1
5	-10.4	-9.4	-0.6	0.9	-23.5	-9.9	-8.6	-0.3	1.0	-21.5	-9.0	-7.7	1.5	2.7	-23.5
7	-10.4	-9.4	-0.8	0.4	-26.2	-10.7	-9.3	-0.7	0.5	-22.6	-9.9	-8.3	0.7	2.2	-23.7
10	-10.7	-9.7	-1.0	0.2	-26.3	-10.3	-9.0	-0.2	1.1	-24.8	-9.8	-8.1	0.4	1.6	-24.4
20	-11.1	-10.1	-1.2	0.0	-27.9	-10.5	-9.2	-0.4	0.9	-26.8	-10.1	-8.5	-0.2	1.1	-25.0
30	-11.2	-10.2	-1.4	0.0	-28.3	-10.3	-9.1	-0.2	1.1	-27.0	-10.1	-8.5	0.2	1.5	-26.7
**No. of Studies**								**Multiplicative**							
2	-0.2	-0.0	5.7	5.6	73.8	0.4	0.4	5.3	5.2	86.5	2.1	2.0	8.5	8.7	86.3
5	-0.1	-0.2	5.2	5.3	67.4	0.7	1.0	6.5	6.5	73.3	0.9	1.0	6.7	6.8	79.8
7	-0.0	-0.1	5.9	6.0	65.0	1.0	1.1	7.1	7.2	71.9	1.3	1.5	7.7	7.8	78.4
10	0.0	0.2	6.0	6.2	63.0	1.0	1.3	7.2	7.2	71.3	1.1	1.0	7.2	7.4	78.4
20	-0.6	-0.6	5.2	5.3	61.3	1.5	1.6	7.3	7.3	69.7	1.1	1.4	7.5	7.5	77.5
30	-0.8	-0.8	5.1	5.3	60.1	1.1	1.3	7.1	7.0	70.0	1.8	1.9	7.8	7.8	76.7
**No. of Studies**								**Recessive**							
2	-89.9	-90.2	-94.2	-95.2	6.4	-88.4	-88.5	-92.8	-94.1	1.2	-88.4	-88.6	-91.9	-93.1	3.2
5	-89.8	-89.9	-93.9	-94.7	-9.3	-88.6	-88.7	-92.4	-93.8	-7.4	-88.2	-88.3	-92.2	-93.6	1.3
7	-89.5	-89.6	-93.4	-94.6	-11.0	-89.4	-89.5	-93.2	-94.3	-7.5	-88.6	-88.7	-92.4	-93.7	-2.6
10	-89.7	-89.7	-93.5	-94.6	-10.6	-89.0	-89.1	-92.9	-94.2	-9.4	-88.1	-88.2	-92.3	-93.6	-4.2
20	-89.0	-89.2	-93.0	-94.3	-10.6	-88.5	-88.7	-92.7	-94.0	-9.2	-88.1	-88.2	-92.5	-93.7	-4.8
30	-89.1	-89.3	-93.1	-94.3	-11.9	-88.3	-88.5	-92.7	-93.9	-9.4	-88.3	-88.5	-92.4	-93.8	-5.5
**No. of Studies**								**Over-dominant**							
2	-19.8	-18.0	-7.0	-4.8	-101	-19.4	-17.5	-6.8	-4.7	-117	-19.9	-17.4	-6.7	-4.0	-116
5	-18.8	-17.1	-5.5	-3.3	-116	-18.6	-16.7	-6.1	-3.7	-117	-19.1	-16.5	-5.7	-3.4	-120
7	-19.3	-17.5	-6.5	-4.4	-117	-19.4	-17.5	-6.5	-3.9	-116	-19.2	-16.9	-6.2	-3.9	-119
10	-20.0	-18.2	-6.7	-4.3	-116	-19.4	-17.3	-6.2	-3.9	-116	-18.8	-16.4	-5.6	-3.4	-119
20	-20.4	-18.7	-7.5	-5.3	-116	-19.2	-17.3	-6.2	-3.8	-118	-19.2	-16.6	-5.9	-3.5	-119
30	-20.5	-18.8	-7.6	-5.3	-116	-19.5	-17.5	-6.3	-3.9	-118	-19.2	-16.6	-5.9	-3.4	-118

Allelic, per-allele odds ratio. LAT, log-additive trend. GOR, generalized odds ratio. Domi, dominant. Rece, recessive. MAF, minor allele frequency. Results are based on 5,000 simulations. τ^2, between-study variance

Table S2. Median percentage bias in the estimated effect size in random-effects meta-analyses using different models of analysis to detect a modest effect (OR = 1.3) of a bi-allelic polymorphism with MAF = 40% that follows distinct modes of action.

Model of analysis	τ^2 =0					τ^2 =0.025					τ^2 =0.05				
	Allelic	LAT	GOR	Domi	Rece	Allelic	LAT	GOR	Domi	Rece	Allelic	LAT	GOR	Domi	Rece
**No. of Studies**						**Dominant**									
2	-42.8	-41.1	-27.6	-0.5	-59.6	-43.5	-41.6	-28.2	-1.5	-60.3	-43.5	-41.4	-27.9	1.3	-61.6
5	-43.0	-41.3	-27.5	-0.5	-59.5	-43.5	-41.9	-28.1	-0.4	-60.6	-43.5	-41.5	-28.0	0.0	-61.4
7	-42.8	-41.3	-27.4	-0.2	-59.3	-43.3	-41.5	-27.8	0.3	-60.9	-43.9	-41.9	-28.5	-0.0	-60.5
10	-43.0	-41.6	-27.6	-0.6	-59.9	-43.2	-41.4	-27.6	0.0	-60.2	-43.8	-41.7	-28.4	0.1	-60.7
20	-42.9	-41.4	-27.5	-0.6	-60.1	-43.4	-41.7	-28.1	-0.8	-60.5	-43.9	-41.9	-28.6	-0.4	-60.9
30	-42.7	-41.2	-27.3	-0.4	-59.9	-43.4	-41.6	-28.0	-0.8	-60.3	-44.2	-42.0	-28.6	-0.7	-61.1
**No. of Studies**						**Multiplicative**									
2	0.1	0.4	19.5	27.0	42.4	-0.5	-0.4	19.1	25.9	40.9	-1.2	-0.8	18.3	26.6	37.8
5	-0.4	-0.2	19.0	26.9	40.3	-0.8	-0.4	18.8	26.4	39.7	-1.1	-0.7	18.5	26.2	40.9
7	-0.2	-0.2	19.1	26.6	40.9	-0.9	-0.7	18.5	26.2	39.2	-0.4	-0.1	19.2	26.4	40.2
10	-0.0	0.0	19.4	27.1	40.4	-1.2	-1.1	18.1	25.8	38.7	-0.9	-0.5	18.8	26.3	40.4
20	-0.1	-0.0	19.3	27.1	39.6	-0.8	-0.6	18.7	26.1	40.3	-0.9	-0.6	18.7	26.7	40.4
30	-0.2	-0.1	19.2	26.9	39.9	-0.7	-0.4	18.9	26.4	40.2	-0.1	0.3	19.7	27.4	40.9
**No. of Studies**						**Recessive**									
2	-57.1	-58.1	-53.2	-72.6	1.1	-56.7	-57.9	-52.6	-72.1	0.5	-56.3	-57.5	-52.3	-72.4	2.9
5	-57.5	-58.7	-53.8	-72.9	0.3	-56.4	-57.7	-52.5	-72.5	0.5	-56.1	-57.5	-52.1	-72.0	2.0
7	-57.5	-58.5	-53.6	-72.8	0.5	-56.7	-57.9	-52.7	-72.2	0.4	-55.6	-57.0	-51.7	-71.8	3.0
10	-57.7	-58.7	-54.0	-73.0	0.1	-56.6	-57.8	-52.7	-72.2	1.1	-55.5	-56.9	-51.6	-71.8	2.6
20	-57.7	-58.8	-53.9	-73.2	-0.3	-56.6	-57.8	-52.6	-72.3	1.0	-55.3	-56.7	-51.2	-71.2	2.2
30	-57.6	-58.7	-53.8	-72.9	-0.6	-56.6	-57.8	-52.7	-72.3	1.1	-55.3	-56.9	-51.5	-71.5	2.3
**No. of Studies**						**Over-dominant**									
2	-79.8	-78.7	-67.7	-22.5	-160	-80.3	-79.0	-68.9	-23.7	-161	-80.5	-79.1	-68.8	-23.2	-162
5	-79.8	-78.7	-68.0	-22.5	-159	-79.7	-78.5	-67.9	-23.0	-159	-79.8	-78.3	-67.7	-22.0	-160
7	-79.7	-78.7	-67.8	-22.3	-159	-79.7	-78.7	-68.0	-23.2	-159	-80.0	-78.8	-68.4	-22.2	-159
10	-79.7	-78.6	-67.9	-22.7	-159	-79.8	-78.6	-68.0	-23.1	-159	-80.2	-78.9	-68.5	-22.6	-160
20	-80.0	-79.0	-68.3	-23.2	-159	-80.1	-79.0	-68.4	-23.2	-159	-80.2	-78.9	-68.6	-22.8	-160
30	-80.1	-79.1	-68.3	-23.2	-159	-80.2	-79.1	-68.5	-22.9	-159	-80.2	-79.0	-68.5	-22.8	-160

Allelic, per-allele odds ratio. LAT, log-additive trend. GOR, generalized odds ratio. Domi, dominant. Rece, recessive. MAF, minor allele frequency. Results are based on 5,000 simulations. τ^2, between-study variance

Table S3. Type-I error rates (%) for the tri-allelic case of fixed-effects meta-analyses according to different genetic models of analysis and heterogeneity (τ^2) for α=5%.

Model of analysis	τ^2=0			τ^2=0.025			τ^2=0.05		
	**Allelic**	**LAT**	**GOR**	**Allelic**	**LAT**	**GOR**	**Allelic**	**LAT**	**GOR**
**No. of Studies**				**$f(A_1)$ = 80%, $f(A_2)$ = 10%, $f(A_3)$ = 10%**					
2	4.46	5	5.46	11.14	7.72	9.56	20.6	11.76	15.54
5	4.72	5.3	5.28	11.64	8.2	10.22	19.94	11.68	15.02
7	4.46	5.12	5.46	11.46	8.04	10.34	20.28	11.18	14.74
10	5.1	5.3	5.42	11.08	8.54	10.28	20.78	11.58	15.52
20	4.84	4.94	5.4	11.46	8.22	11.08	21.2	12.72	16.6
30	4.4	4.98	5.1	12.18	9.02	10.98	22.56	13.86	18.12
**No. of Studies**				**$f(A_1)$ = 50%, $f(A_2)$ = 10%, $f(A_3)$ = 40%**					
2	4.06	5.04	5.06	11.14	10.44	9.14	18.32	14.22	11.8
5	3.74	4.38	4.28	11.26	10.22	9.4	18.94	15	12.6
7	3.48	4.72	4.66	11.64	10.24	9.12	18.98	14.52	12.82
10	3.58	4.4	4.52	10.76	10	9.04	18.86	15.32	13.36
20	3.7	4.52	4.6	11.64	9.76	8.82	19.6	15.4	14.06
30	3.74	4.38	4.76	11.88	10.28	9.66	20.16	16	14.26
**No. of Studies**				**$f(A_1)$ = 20%, $f(A_2)$ = 40%, $f(A_3)$ = 40%**					
2	4.28	5.06	5.02	11.15	9.22	9.50	16.26	11.50	11.70
5	4.26	4.78	4.92	8.18	7.18	7.24	11.02	8.88	9.21
7	4.42	5	4.92	7.76	6.59	6.63	8.45	7.36	7.38
10	4.34	4.92	5.02	6.50	6.33	6.31	7.02	6.21	6.19
20	4.3	5.26	5.3	6.85	5.94	5.91	6.71	5.94	6.07
30	4.14	5.44	5.44	11.15	9.22	9.50	16.26	11.50	11.70
**No. of Studies**				**$f(A_1)$ = 33.3%, $f(A_2)$ = 33.3%, $f(A_3)$ = 33.3%**					
2	4.84	5.32	5.16	9.42	7.96	8.10	23.8	17.84	17.74
5	4.5	5.06	5.04	8.00	7.22	7.26	23.12	18.72	18.78
7	4.54	5.2	5.2	7.32	6.90	6.98	23.42	18.26	18.22
10	4.56	4.96	5.02	6.02	5.78	5.90	23.3	18.36	18.32
20	4.7	5.12	5.26	6.10	5.74	5.80	24.7	19.6	19.64
30	4.58	5.38	5.5	9.42	7.96	8.10	24.44	18.62	18.78

Allelic, per-allele odds ratio. LAT, log-additive trend. GOR, generalized odds ratio. Results are based on 5,000 simulations. τ^2**,** between-study variance. Estimates were computed having the allele A_1 as reference. *f*, allelic frequency.

Table S4. Type-I error rates (%) for the tri-allelic case of random-effects meta-analyses according to different genetic models of analysis and heterogeneity (τ^2) for α =5%.

Model of analysis	τ^2 =0			τ^2 =0.025			τ^2 =0.05		
	Allelic	LAT	GOR	Allelic	LAT	GOR	Allelic	LAT	GOR
**No. of Studies**				**$f(A_1)$ = 80%, $f(A_2)$ = 10%, $f(A_3)$ = 10%**					
2	3.44	3.88	4.2	7.7	5.38	6.44	14.06	8.2	10.48
5	3.34	4.24	4.24	7.04	5.54	6.62	10.02	7.5	8.78
7	3.4	4.18	4.36	6.32	5.28	6.44	9.78	6.78	7.94
10	4.02	4.52	4.46	6.2	5.76	6.62	9.18	6.88	7.68
20	3.64	4.16	4.52	5.68	5.28	6.42	7.9	7.28	7.28
30	3.48	4.26	4.58	5.54	6.22	6.86	7.44	7.78	8.02
**No. of Studies**				**$f(A_1)$ = 50%, $f(A_2)$ = 10%, $f(A_3)$ = 40%**					
2	3.16	4	3.98	8.26	7.7	6.7	11.74	8.9	7.66
5	2.78	3.2	3.28	6.72	6.54	6.26	9.4	7.46	6.8
7	2.36	3.72	3.64	7.02	6.66	6.06	8.16	7.4	6.98
10	2.44	3.44	3.6	6.08	6.1	5.84	7.3	6.7	6.62
20	2.88	3.88	3.74	6.02	5.58	5.38	6.06	6.86	6.84
30	3.08	3.74	4	5.98	5.82	6.26	6.2	6.64	6.68
**No. of Studies**				**$f(A_1)$ = 20%, $f(A_2)$ = 40%, $f(A_3)$ = 40%**					
2	3.24	4.02	3.96	18.92	17.48	17.74	26.34	22.4	22.68
5	3	3.66	3.82	16.24	15.54	15.74	22.12	19.76	19.98
7	3.04	3.92	3.92	16.06	15.28	15.36	20.82	18.96	19.08
10	3.3	3.88	3.98	15.34	15.18	15.16	19.84	18.24	18.46
20	3.42	4.36	4.28	14.64	14.56	14.48	17.78	17	17.2
30	3.22	4.58	4.52	14.26	13.9	13.86	16.66	16.56	16.6
**No. of Studies**				**$f(A_1)$ = 33.3%, $f(A_2)$ = 33.3%, $f(A_3)$ = 33.3%**					
2	3.6	4.18	4.06	9.74	8.14	8.28	16.12	11.02	10.96
5	3.34	3.76	3.82	8.14	7.34	7.48	10.62	8.84	9.02
7	3.54	4	3.98	7.06	7.24	7.34	9.96	8.24	8.2
10	3.5	4.02	4.02	6.96	6.92	7.16	9.24	7.48	7.36
20	3.66	3.96	4.08	6.14	6.38	6.34	6.86	6.7	6.72
30	3.42	4.34	4.38	6.08	5.56	5.78	6.48	6.3	6.32

Allelic, per-allele odds ratio. LAT, log-additive trend. GOR, generalized odds ratio. Results are based on 5,000 simulations. τ^2, between-study variance. Estimates were computed having the allele A_1 as reference. *f*, allelic frequency.

Table S5. Empirical power (%) for the tri-allelic case of random-effects meta-analyses according to different genetic models of analysis and heterogeneity (τ^2) for α=5% when two alleles modulate the risk of disease in opposite directions (Dominant effects, OR[A_2] = 0.77 and OR[A_3]=1.3), but prior evidence on the direction of effects is available.

Model of analysis	τ^2=0			τ^2=0.025			τ^2=0.05		
	**Allelic**	**LAT**	**GOR**	**Allelic**	**LAT**	**GOR**	**Allelic**	**LAT**	**GOR**
**No. of Studies**				**$f(A_1)$ = 80%, $f(A_2)$ = 10%, $f(A_3)$ = 10%**					
2	63.11	69.09	71.56	57.68	57.32	59.39	55.28	51.92	53.33
5	96.59	98.27	98.82	86.25	88.50	89.59	76.91	78.80	80.69
10	100	100	100	98.81	99.36	99.46	94.99	96.70	97.51
20	100	100	100	100	100	100	99.94	99.94	99.96
30	100	100	100	100	100	100	100	100	100
**No. of Studies**				**$f(A_1)$ = 50%, $f(A_2)$ = 40%, $f(A_3)$ = 10%**					
2	73.12	79.74	78.32	64.06	64.84	64.88	55.10	55.00	54.56
5	98.48	99.48	99.32	89.84	93.78	93.50	77.54	83.66	83.56
10	100	100	100	99.40	99.78	99.80	96.36	98.36	98.06
20	100	100	100	100	100	100	99.96	99.96	99.94
30	100	100	100	100	100	100	100	100	100
**No. of Studies**				**$f(A_1)$ = 50%, $f(A_2)$ = 10%, $f(A_3)$ = 40%**					
2	62.52	76.82	74.22	56.40	61.14	59.74	51.50	51.94	51.50
5	95.12	99.24	98.74	85.02	90.82	89.86	74.04	78.28	77.24
10	99.92	100	100	99.02	99.72	99.58	94.02	96.72	96.14
20	100	100	100	100	100	100	99.98	100	100
30	100	100	100	100	100	100	100	100	100
**No. of Studies**				**$f(A_1)$ = 20%,$f(A_2)$ = 40%, $f(A_3)$ = 40%**					
2	84.36	89.58	89.82	67.72	70.45	71.80	58.62	60.49	61.43
5	99.62	99.98	99.96	92.02	95.72	95.89	81.25	86.84	87.22
10	100	100	100	99.90	100	100	97.82	98.98	98.98
20	100	100	100	100	100	100	100	100	100
30	100	100	100	100	100	100	100	100	100
**No. of Studies**				**$f(A_1)$ = 33.3%,$f(A_2)$ = 33.3%, $f(A_3)$ = 33.3%**					
2	85.44	90.46	90.74	69.16	71.74	72.26	69.16	71.74	72.26
5	99.88	100	99.98	92.74	95.76	95.84	92.74	95.76	95.84
10	100	100	100	99.84	99.98	99.98	99.84	99.98	99.98
20	100	100	100	100	100	100	100	100	100
30	100	100	100	100	100	100	100	100	100

Allelic, per-allele odds ratio. LAT, log-additive trend. GOR, generalized odds ratio. *f*, allele frequency. Results are based on 5,000 simulations. τ^2**,** between-study variance. Estimates were computed having the allele A_1 as reference.

Table S6. Empirical power (%) for the tri-allelic case of random-effects meta-analyses according to different genetic models of analysis and heterogeneity (τ^2) for α=5% when two alleles modulate the risk of disease in opposite directions (Multiplicative effects, OR[A_2] = 0.77 and OR[A_3]=1.3), but prior evidence on the direction of effects is available.

Model of analysis	τ^2 =0			τ^2 =0.025			τ^2 =0.05		
	**Allelic**	**LAT**	**GOR**	**Allelic**	**LAT**	**GOR**	**Allelic**	**LAT**	**GOR**
**No. of Studies**				***f*(A_1) = 80%, *f*(A_2) = 10%, *f*(A_3) = 10%**					
2	73.04	76.52	76.68	62.6	60.26	61.14	58.28	52.58	53.62
5	98.58	99.1	99.26	90.4	91.12	91.6	79.68	79.82	81.58
10	99.9	99.94	99.94	96.46	97.18	97.72	89.32	90.98	92.1
20	100	99.98	100	99.44	99.56	99.72	96.28	97.2	97.72
30	100	100	100	100	100	100	99.98	99.98	100
**No. of Studies**				***f*(A_1) = 50%, *f*(A_2) = 40%, *f*(A_3) = 10%**					
2	93.8	94.8	95.1	77.56	74.64	74.24	70.28	64.9	63.66
5	100	100	100	95.7	97.42	97	87.44	88.4	87.26
10	100	100	100	99.2	99.64	99.56	93.94	95.64	95.08
20	100	100	100	99.9	99.98	99.98	98.7	99.3	99.24
30	100	100	100	100	100	100	100	100	100
**No. of Studies**				***f*(A_1) = 50%, *f*(A_2) = 10%, *f*(A_3) = 40%**					
2	92.02	93.66	94.2	74.72	70.2	70.18	67.94	57.34	57.24
5	99.92	100	100	94.86	94.98	94.74	84.66	82.58	82.26
10	100	100	100	98.72	99.16	99	92.3	92.38	92.1
20	100	100	100	99.88	99.96	99.94	97.8	98.06	97.92
30	100	100	100	100	100	100	100	99.98	99.98
**No. of Studies**				***f*(A_1) = 20%, *f*(A_2) = 40%, *f*(A_3) = 40%**					
2	98.78	99.12	99.18	85.14	82.70	82.94	76.07	69.52	69.75
5	100	100	100	98.60	99.16	99.16	91.24	93.23	93.23
10	100	100	100	100	100	100	99.52	99.74	99.71
20	100	100	100	100	100	100	100	100	100
30	100	100	100	100	100	100	100	100	100
**No. of Studies**				***f*(A_1) = 33.3%, *f*(A_2) = 33.3%, *f*(A_3) = 33.3%**					
2	98.7	98.72	98.72	83.6	79.72	79.92	74.1	65.78	66
5	100	100	100	97.66	98.34	98.36	88.68	90.62	90.64
10	100	100	100	99.72	99.84	99.86	99.30	99.56	99.56
20	100	100	100	99.98	100	100	100	100	100
30	100	100	100	100	100	100	100	100	100

Allelic, per-allele odds ratio. LAT, log-additive trend. GOR, generalized odds ratio. *f*, allele frequency. Results are based on 5,000 simulations. τ^2, between-study variance. Estimates were computed having the allele A_1 as reference.

Table S7. Empirical power (%) for the tri-allelic case of random-effects meta-analyses according to different genetic models of analysis and heterogeneity (τ^2) for α=5% when two alleles modulate the risk of disease in opposite directions (Recessive effects, OR[A_2] = 0.77 and OR[A_3]=1.3), but prior evidence on the direction of effects is available

Model of analysis	τ^2=0			τ^2=0.025			τ^2=0.05		
	Allelic	LAT	GOR	Allelic	LAT	GOR	Allelic	LAT	GOR
**No. of Studies**				**$f(A_1)$ = 80%, $f(A_2)$ = 10%, $f(A_3)$ = 10%**					
2	4.27	5.02	4.19	4.35	4.75	4.31	3.99	4.11	4.05
5	4.31	5.32	4.47	5.15	5.57	4.69	5.06	5.26	4.66
10	6.68	7.24	5.80	6.52	7.46	5.94	6.64	7.85	6.48
20	10.95	11.47	8.20	11.11	11.87	7.96	10.58	11.76	8.87
30	15.47	16.15	11.33	15.70	15.92	10.77	15.67	16.07	11.20
**No. of Studies**				**$f(A_1)$ = 50%, $f(A_2)$ = 40%, $f(A_3)$ = 10%**					
2	14.50	15.94	18.60	16.54	17.92	19.60	16.74	17.56	19.40
5	36.60	38.22	44.18	33.52	35.12	39.40	32.92	33.34	36.08
10	70.98	67.50	74.22	62.38	62.22	67.08	55.10	56.04	58.88
20	96.20	94.28	96.68	91.68	90.64	93.42	83.68	83.94	86.06
30	99.72	99.14	99.72	98.52	98.22	99.02	95.66	95.38	96.46
**No. of Studies**				**$f(A_1)$ = 50%, $f(A_2)$ = 10%, $f(A_3)$ = 40%**					
2	18.32	18.80	22.56	20.04	20.48	23.32	22.00	22.14	25.38
5	45.72	44.64	51.60	40.96	40.88	46.72	39.06	40.92	44.50
10	79.88	76.88	83.92	70.84	69.38	76.50	63.12	64.40	69.16
20	98.64	97.16	98.88	95.86	95.00	97.18	90.70	91.08	93.68
30	99.94	99.82	99.96	99.72	99.52	99.82	98.58	98.20	98.84
**No. of Studies**				**$f(A_1)$ = 20%, $f(A_2)$ = 40%, $f(A_3)$ = 40%**					
2	49.52	57.44	57.38	45.23	49.17	49.63	41.55	44.96	45.19
5	90.48	93.70	93.54	76.94	82.32	82.07	67.09	72.56	72.13
10	99.86	99.92	99.92	97.26	98.40	98.52	91.63	94.84	94.84
20	100	100	100	100	100	100	99.77	99.84	99.84
30	100	100	100	100	100	100	100	100	100
**No. of Studies**				**$f(A_1)$ = 33.3%, $f(A_2)$ = 33.3%, $f(A_3)$ = 33.3%**					
2	30.80	35.02	34.32	31.34	33.64	32.92	32.26	33.36	33.08
5	69.74	72.60	71.42	62.06	66.16	65.28	57.76	61.34	60.76
10	95.82	96.18	95.98	91.06	92.46	92.16	84.74	87.52	86.88
20	99.96	99.94	99.94	99.80	99.86	99.84	98.90	99.30	99.26
30	100	100	100	100	100	100	99.98	99.98	99.98

Allelic, per-allele odds ratio. LAT, log-additive trend. GOR, generalized odds ratio. *f*, allele frequency. Results are based on 5,000 simulations. τ^2**,** between-study variance. Estimates were computed having the allele A_1 as reference.

Table S8. Empirical power (%) for the tri-allelic case of random-effects meta-analyses according to different genetic models of analysis and heterogeneity (τ^2) for α =5% when only a single allele modulates the risk of disease (Dominant effects, OR[A_3]=1.3)

	τ^2 =0			τ^2 =0.025			τ^2 =0.05		
**Model of analysis**	**Allelic**	**LAT**	**GOR**	**Allelic**	**LAT**	**GOR**	**Allelic**	**LAT**	**GOR**
**No. of Studies**				***f*(A_1) = 80%, *f* (A_2) = 10%, *f* (A_3) = 10%**					
2	31.58	31.02	29.6	31.54	31.26	30.26	30.78	29.32	28.66
5	72.48	67.34	65.08	58.24	56.98	55.78	48.16	48.1	47.76
10	96.96	94.36	92.78	85.22	85	83.92	73.86	75.36	74.4
20	99.96	99.86	99.84	99.04	98.86	98.7	95.8	96.88	96.46
30	100	100	100	99.94	99.98	99.9	99.42	99.56	99.6
**No. of Studies**				***f*(A_1) = 50%, *f* (A_2) = 40%, *f* (A_3) = 10%**					
2	33.2	18.7	17.66	33.38	19.44	18.96	31.34	20.16	19.86
5	74.04	41.58	40.2	58.72	37.7	36.62	47.4	35.68	34.56
10	97.24	72.24	70.2	86.7	65.1	63.3	72.64	58.54	58.02
20	99.98	95.74	94.84	99.22	92.42	91.52	95.28	87.82	87.12
30	100	99.52	99.42	99.98	98.88	98.7	99.48	96.86	96.66
**No. of Studies**				***f*(A_1) = 50%, *f* (A_2) = 10%, *f* (A_3) = 40%**					
2	29.98	33.84	41.1	30.28	32.10	37.30	30.46	31.64	35.32
5	71.96	72.02	81.32	56.74	59.62	65.60	47.60	50.50	54.76
10	96.56	95.76	98.5	84.50	85.56	89.96	72.92	76.28	80.48
20	100	99.98	100	99.00	98.98	99.54	94.70	96.26	97.40
30	100	100	100	99.92	99.88	99.96	99.44	99.42	99.66
**No. of Studies**				***f*(A_1) = 20%, *f* (A_2) = 40%, *f* (A_3) = 40%**					
2	33.06	28.26	28.72	30.18	25.72	26.22	31.94	27.2	27.88
5	72.32	60.28	61.94	58.12	50.42	51.26	49.94	45.72	45.94
10	96.96	90.7	91.38	85.66	79.32	80.42	74.78	71.14	71.82
20	99.98	99.74	99.82	99.24	97.54	97.92	95.68	93.9	94.14
30	100	100	100	99.9	99.68	99.68	99.34	99.18	99.28
**No. of Studies**				***f*(A_1) = 33.3%, *f* (A_2) = 33.3%, *f* (A_3) = 33.3%**					
2	38.66	31.7	33.38	36.1	30.58	31.88	33.96	28.76	29.72
5	79.64	68.32	71.26	62.96	56.18	57.54	52	47.28	48.56
10	98.64	94.32	95.62	89.24	84.72	85.8	76.64	74.4	75.44
20	100	99.96	99.96	99.58	98.88	99.02	96.24	95.56	96.02
30	100	100	100	100	99.84	99.92	99.72	99.48	99.56

Allelic, per-allele odds ratio. LAT, log-additive trend-based odds ratio. GOR, generalized odds ratio. *f*, allele frequency. Results are based on 5,000 simulations. τ^2**,** between-study variance. Estimates were computed having the allele A_1 as reference.

Table S9. Empirical power (%) for the tri-allelic case of random-effects meta-analyses according to different genetic models of analysis and heterogeneity (τ^2) for α =5% when only a single allele modulates the risk of disease (Multiplicative effects, OR[A_3]=1.3)

	τ^2 =0			τ^2 =0.025			τ^2 =0.05		
**Model of analysis**	**Allelic**	**LAT**	**GOR**	**Allelic**	**LAT**	**GOR**	**Allelic**	**LAT**	**GOR**
**No. of Studies**				**$f(A_1)$ = 80%, $f(A_2)$ = 10%, $f(A_3)$ = 10%**					
2	40.28	39.26	34.08	37.86	37.54	33.82	33.68	33.48	30.7
5	81.22	78.94	71.52	64.3	65.68	60.68	51.74	53.94	50.2
10	98.86	98.22	96.24	89.62	90.7	87.12	76.3	80.34	77.58
20	100	100	99.98	99.6	99.58	99.32	97.04	97.72	97.04
30	100	100	100	99.98	99.98	99.92	99.66	99.78	99.6
**No. of Studies**				**$f(A_1)$ = 50%, $f(A_2)$ = 40%, $f(A_3)$ = 10%**					
2	40.4	22.42	20.18	36.22	23.16	21.68	34.82	24.92	23.24
5	82.86	51.1	46.16	62.98	43.92	40.7	52.24	43.24	40.58
10	99.28	83.48	78.54	89.76	73.36	70.28	77.36	67.84	65.28
20	100	98.98	97.94	99.62	95.82	94.02	96.92	92.5	90.66
30	100	99.94	99.88	100	99.52	99.24	99.76	98.62	98.1
**No. of Studies**				**$f(A_1)$ = 50%, $f(A_2)$ = 10%, $f(A_3)$ = 40%**					
2	76.06	80.24	81.38	54.52	57.36	57.06	46.98	47.46	47.72
5	99.4	99.52	99.54	80.14	83.34	83.34	61.78	66.62	67.02
10	100	100	100	96.86	98.34	98.54	85.74	90.52	90.92
20	100	100	100	100	99.98	100	99.08	99.68	99.72
30	100	100	100	100	100	100	99.96	99.98	100
**No. of Studies**				**$f(A_1)$ = 20%, $f(A_2)$ = 40%, $f(A_3)$ = 40%**					
2	77.12	65.86	71	56.82	50.78	52.52	51.2	49.3	50.04
5	99.38	97.18	98.36	80.86	78.34	80.08	66.2	67.54	68.76
10	100	100	100	96.94	96.58	97.22	87.56	89.74	90.72
20	100	100	100	99.98	99.98	99.98	99.34	99.54	99.64
30	100	100	100	100	100	100	99.98	100	100
**No. of Studies**				**$f(A_1)$ = 33.3%, $f(A_2)$ = 33.3%, $f(A_3)$ = 33.3%**					
2	76.48	66.06	67.22	54.68	49.28	49.72	45.42	41.6	41.58
5	99.38	96.84	97.14	78.66	77.82	78.06	61.52	63.3	63.5
10	100	99.96	99.98	97.28	97.08	97.28	85.38	88.1	88.42
20	100	100	100	100	100	100	98.9	99.18	99.24
30	100	100	100	100	100	100	99.94	100	100

Allelic, per-allele odds ratio. LAT, log-additive trend-based odds ratio. GOR, generalized odds ratio. *f*, minor allele frequency. Results are based on 5,000 simulations. τ^2**,** between-study variance. Estimates were computed having the allele A_1 as reference.

Table S10. Empirical power (%) for the tri-allelic case of random-effects meta-analyses according to different genetic models of analysis and heterogeneity (τ^2) for α =5% when only a single allele modulates the risk of disease (Recessive effects, OR[A_3]=1.3)

Model of analysis	τ^2 =0			τ^2 =0.025			τ^2 =0.05		
	**Allelic**	**LAT**	**GOR**	**Allelic**	**LAT**	**GOR**	**Allelic**	**LAT**	**GOR**
**No. of Studies**				**$f(A_1)$ = 80%, $f(A_2)$ = 10%, f (A_3) = 10%**					
2	3.47	4.17	3.81	3.48	4.35	4.33	3.24	4.04	3.72
5	3.73	4.81	3.65	4.27	5.23	4.33	3.86	4.93	3.54
10	4.35	6.21	4.03	4.93	6.95	4.71	4.57	7.16	4.89
20	5.89	9.84	5.21	7.57	10.05	5.69	7.16	10.46	5.27
30	7.78	13.03	5.85	9.03	13.52	5.69	9.47	13.52	6.08
**No. of Studies**				**$f(A_1)$ = 50%, f (A_2) = 40%, f (A_3) = 10%**					
2	3.12	4.24	3.92	3.88	3.92	3.76	4.26	4.14	3.90
5	3.74	4.22	4.18	4.50	4.44	4.12	4.24	4.46	4.04
10	5.12	5.34	4.76	4.60	4.38	3.70	4.80	5.24	4.32
20	6.76	6.52	5.38	7.14	6.76	5.02	7.52	6.82	4.90
30	9.06	8.08	6.24	9.72	7.90	6.00	10.06	8.04	5.72
**No. of Studies**				**$f(A_1)$ = 50%, f (A_2) = 10%, f (A_3) = 40%**					
2	16.54	25.94	20.78	18.90	26.26	22.24	20.90	25.62	22.56
5	41.52	56.88	47.02	38.02	49.54	42.80	35.30	44.36	39.48
10	77.06	87.40	79.24	66.52	78.52	70.54	60.06	71.08	65.72
20	97.88	99.40	97.76	93.38	97.44	94.22	88.78	94.58	91.54
30	99.92	100	99.78	99.08	99.72	99.24	97.78	99.14	98.16
**No. of Studies**				**fA_1) = 20%, f (A_2) = 40%, f (A_3) = 40%**					
2	17.82	17.00	20.04	20.34	18.68	21.94	22.06	20.06	23.00
5	44.50	39.14	47.28	40.14	36.34	41.70	38.25	35.90	40.73
10	76.84	68.98	77.90	68.52	62.64	69.92	62.17	59.37	65.12
20	97.96	94.26	97.36	94.08	90.02	94.12	89.69	85.58	90.21
30	99.92	99.22	99.80	99.18	97.92	99.12	97.69	96.37	98.05
**No. of Studies**				**$f(A_1)$ = 33.3%, f (A_2) = 33.3%, f (A_3) = 33.3%**					
2	13.32	13.52	13.12	14.82	14.42	13.72	14.82	14.42	13.72
5	30.04	28.18	27.12	29.48	28.22	27.02	29.48	28.22	27.02
10	57.56	53.36	51.40	52.70	50.50	48.34	52.70	50.50	48.34
20	89.26	84.78	82.92	84.52	80.60	78.56	84.52	80.60	78.56
30	98.66	96.34	95.30	96.28	93.60	92.60	96.28	93.60	92.60

Allelic, per-allele odds ratio. LAT, log-additive trend-based odds ratio. GOR, generalized odds ratio. *f*, minor allele frequency. Results are based on 5,000 simulations. τ^2**,** between-study variance. Estimates were computed having the allele A_1 as reference.
